# Research on the Effects of Lying on Memory: A Scientometric Analysis and a Call for New Studies

**DOI:** 10.3389/fpsyg.2022.837265

**Published:** 2022-02-24

**Authors:** Fabiana Battista, Henry Otgaar

**Affiliations:** ^1^Leuven Institute of Criminology, Catholic University of Leuven, Leuven, Belgium; ^2^Department of Clinical Psychological Science, Maastricht University, Maastricht, Netherlands

**Keywords:** lying, memory, scientometric analyses, review, future direction

## Abstract

Research on the effects of lying and memory is increasingly attracting empirical attention. In the current manuscript, a scientometric analysis was carried out on the mnemonic consequences of lying. This analysis took into account 70 published articles extracted from Scopus and Web of Science databases from 1998 to 2021. A scientometric analysis was conducted in order to visualize the state of the art on this line of research (i.e., authors, countries, institutions, journals, and co-citations). Additionally, a keywords’ cluster analysis was executed to investigate the main keywords used in the published papers. Based on the keywords’ cluster analysis, we identified the main aims and critical issues of the reviewed papers. The United States and the Netherlands are the two most productive countries into the effects of lying on memory. The top five authors are mainly from European countries and wrote from 6 to 15 articles. The cluster analysis detected three clusters of keywords. The critical issues of this line of research are mainly related to the generalizability of the achieved findings for real situations, a lack of a direct control of the manipulation adopted, and a need of additional measures. The current analysis provides a comprehensive overview and understanding of existing research on the effects of lying on memory and provides possible future directions of this research domain.

## Introduction

It has been widely established that several factors (e.g., cognitive differences, stress, and time) can affect our memory for an event (e.g., Loftus, 2005). Recently, a new line of research is emerging showing that memory can also be contaminated due to the act of lying. This work has shown that memory is differentially affected depending on the type of lie that is executed (e.g., false denial, feigning amnesia, fabricating; e.g., Pickel, 2004; Chrobak and Zaragoza, 2008; Vieira and Lane, 2013; Otgaar et al., 2016; Mangiulli et al., 2018; Romeo et al., 2019; Battista et al., 2020, 2021b; Li and Liu, 2021).

Lying can be defined as “a successful or unsuccessful deliberate attempt, without forewarning, to create in another a belief which the communicator considers to be untrue” (Vrij, 2008, p. 15). Based on this definition, it has been argued that during lying, the truth needs to be inhibited and another and believable alternative needs to be provided (e.g., Vrij, 2008). The act of lying can occur in several different ways. Specifically, people can falsely deny, simulate amnesia, and/or fabricate an alternative story (e.g., Goodman-Brown et al., 2003; Pyszora et al., 2003; Bourget and Whitehurst, 2007; Chrobak and Zaragoza, 2008; Block et al., 2012; O’Donohue et al., 2018). Importantly, the type of deceptive strategy can have different impact on memory. Specifically, false denials and feigning amnesia have shown to lead to forgetting while fabrication leads to the formation of commission errors^1^ (Otgaar and Baker, 2018).

## Studying the Effects of Lying on Memory

Scholars have used different paradigms to examine the impact of lying on memory. For example, in studies on false denials (e.g., Vieira and Lane, 2013; Otgaar et al., 2016, 2018; Battista et al., 2021a; Li and Liu, 2021) researchers typically ask participants to watch some pictures or a video (i.e., mock crime) and subsequently require them to answer some questions regarding the stimulus (i.e., pictures or video) either without guessing or denying the occurrence of several experienced detail. After an interval, participants’ memory is tested through a source monitoring task and/or cued questions with the instructions to provide an honest response for all questions. In some studies, participants’ memory is tested in terms of memory for the stimulus but also in terms of memory for the interview performed during the first session. Here, the recurrent finding is a memory undermining effect for the interview, termed *denial-induced forgetting* (DIF; Otgaar et al., 2016; but see also Li and Liu, 2021). That is, people who denied the occurrence of stimulus-related details are less likely to remember whether they discussed these details during the interview than people who told the truth. A few studies (e.g., Romeo et al., 2018; Battista et al., 2021a) have also shown that, in certain specific circumstances, false denials can also undermine the memory for the event. This seems to occur when the act of denials requires a high involvement, such as an emotional and active involvement (e.g., Romeo et al., 2018) or higher cognitive effort to perform the lie (e.g., Battista et al., 2020).

In studies on feigning amnesia, participants are asked to read a narrative (e.g., Bylin and Christianson, 2002) or watch a mock crime video (e.g., Mangiulli et al., 2019a,b) by identifying themselves as the offender of the crime. Then, participants are asked to either tell the truth concerning the crime or feign memory loss. A week later, all participants are invited to tell the truth regarding the crime. In general, these studies have revealed that feigning amnesia leads to impoverished recall of the crime (e.g., Christianson and Bylin, 1999; Van Oorsouw and Merckelbach, 2004; Van Oorsouw and Giesbrecht, 2008; Mangiulli et al., 2019a,b).

Moreover, scholars have found that feigning amnesia can lead to both omissions and commissions depending on the way in which the liar has simulated amnesia (Otgaar and Baker, 2018). That is, if the liar uses a simple lie (e.g., *I do not remember what happened*), the act of feigning amnesia leads to omissions, while if the liar uses a more elaborated lie (e.g., *I do not remember what happened because I was busy with my son*), feigning amnesia has been shown to elevate commission errors.

Finally, studies on the effects of fabrication on memory have frequently used the *forced confabulation paradigm* (e.g., Ackil and Zaragoza, 1998, 2011; Zaragoza et al., 2001). Overall, studies using this paradigm (e.g., Chrobak and Zaragoza, 2008, 2012; Battista et al., 2021b) followed this procedure: Participants watch a video and then reply to some questions regarding the stimulus. A group of them is instructed to honestly answer these questions while a second group has to confabulate an answer. After an interval, participants’ memory for the event is assessed. The typical finding here is that people who fabricated details recall their own self-generated detail as true details of the event. In other words, this deceptive strategy leads to commission errors.

Another effect related the effect of fabrication on memory is a phenomenon called the *fabrication inflation* effect (Polage, 2004, 2012). That is, in these studies (Polage, 2004, 2012, 2018) it was examined whether fabricating an alternative story of the original event can affect people’s beliefs in the occurrence of such a fabricated story. Indeed, in these studies, participants -after rating which events were highly unlikely to have been experienced – are invited to provide a false version of the event that was indicated to being never experienced by them (i.e., fabricated experimental event). After a delay (e.g., 1 or 5 weeks), participants are instructed to provide a honest response and rate for a second time beliefs about the occurrence of all the events (i.e., fabricated experimental event vs. control events). She found that a subsample of participants (around 15% of the total sample) increased their beliefs in the occurrence of the fabricated event compared with the control events.

Otgaar and Baker (2018) proposed the Memory and Deception (MAD) framework to explain the memory outcomes of each deceptive strategy on memory and the possible underlying mechanisms associated with these outcomes. Based on the available literature, they concluded that false denials lead to omissions, feigning amnesia to omissions and commission errors, and fabrication to commission errors. Otgaar and Baker put forward the idea that these different memory effects on memory could be due to the amount of cognitive resources used during the act of lying. In particular, they suggested that each deceptive strategy requires a different amount of cognitive resources and these differences in cognitive resources might underlie the respective outcomes on memory. Therefore, the false denials strategy is assumed to be the simplest strategy and fabrication the most complex one. Furthermore, they postulated that the effects of false denials on memory could also be caused to mechanisms such as inhibition underpinning classical forgetting effects, like the directing forgetting (Basden et al., 1993), retrieval-induced forgetting (Anderson et al., 1994), and forgetting caused by the Think/No Think paradigm (Anderson and Green, 2001). Also, for the memory undermining effect of feigning amnesia they argued that a possible explanation could be a lack of rehearsal (e.g., Christianson and Bylin, 1999; Van Oorsouw and Merckelbach, 2004) of the original information or a source monitoring confusion (Johnson et al., 1993) between false and true information. Relatedly, source monitoring errors were regarded as also the most likely explanation underlying the effects of fabrication on memory.

## A Scientometric Analysis

There are several ways to increase our understanding on the effects of lying on memory. One way is to build on previous studies by changing several manipulations thereby examining how these manifest in the effects of lying on memory. Another way is to meta-analytically examine the strengths of the effects of lying on memory. The principal method that we used in the current manuscript was to perform a scientometric analysis to understand the main themes and research questions investigated in studies on lying and memory. The method of scientometric analysis was first defined by Mulchenko (1969) as a quantitative technique to assess the evolution of research. More recently, Hook and Börner (2005) added that scientometric analyses allow researchers to have “the graphic rendering of bibliometric data designed to provide a global view of a particular domain, the structural details of a domain, the salient characteristics of a domain (its dynamics, most cited authors or papers, bursting concepts, etc.) or all three” (p. 201). In other words, scientometric analyses permit researchers to assess the development of a specific research line by assessing authors’ and journals’ contribution and the impact of publications and to identify the main themes associated with such a specific research domain (Börner et al., 2003; Su and Lee, 2010; Mao et al., 2015; Caffò et al., 2020).

Certain scientometric analyses have also been performed in the area of memory. For instance, Dodier (2019) conducted a bibliometric analysis in the field of recovered memories. The author conducted a series of analyses on research published in the 21st century by using bibliometric information, such as the year of publications, the authors, the country/region of authors, the name of the journals, the number of citations, and keywords. His analysis provides an example of the advantages the findings of such analyses can offer to researchers and a picture of the evaluation of research on repressed and recovered memories. Indeed, the results underlined that the topic captured the attention of several scholars, in different countries, and in different research fields (i.e., clinical and cognitive researchers). In addition, he gave information on the content of articles showing that the debate on the occurrence of recovered and repressed memories remained still stable. Based on these results, the author was able to provide interesting and useful insights for upcoming research by suggesting the need of further investigation on repressed and recovered memories and the need for practitioners (e.g., legal, clinicians) to be aware that the debate surrounding the topic is not over. Similarly, we first wanted to detect the evolution of research in the area of lying and memory. Second, our aim was to identify which critical elements are missing in the area of lying and memory. Specifically, by doing a scientometric analysis, we wanted to collect and provide information (i) on the performance of countries, institutions, and authors to recognize the main actors in this line of research and (ii) on the main challenges and issues addressed by researchers with the aim to highlight possible future research directions. In order to address this second aim, we followed a more traditional review approach by conducting additional analyses on the most representative publications on lying and memory.

## Method

### Data Collection

**FIGURE 1:**
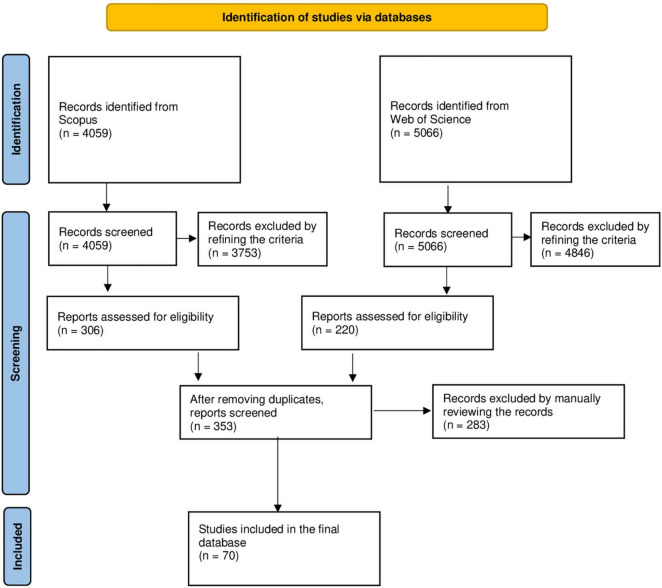
The PRISMA chart showing the selection of the publications.

The search of relevant publications was performed by using two databases: Scopus and Web of Science (WoS).^2^ These two databases have been chosen because they are the largest abstract and citations databases of peer-reviewed and multidisciplinary publications (Guz and Rushchitsky, 2009). The literature search on both Scopus and WoS was carried out on October 21st, 2021. No range of years was selected in order to collect all the publications on the topic. For both databases, the search code for retrieving publications was: “Lying and Memory” OR “Deception and Memory” OR “Deceptive Strategies and Memory” OR “False Denials and Memory” OR “Feigned Amnesia and Memory” OR “Simulation of Amnesia and Memory” OR “Fabrication and Memory” OR “Forced Confabulation and Memory” in the “Title, Abstract, Keywords” search in Scopus database and in “Topic” search in WoS. In this way, it was possible to retrieve the following information: Title, Abstract, Authors, Keywords, Keywords Plus, Authors’ Information (i.e., country, address, e-mail, ORCID), Publication Information (i.e., journal, date of publication, volume, issue, doi, total citations), and Journal Information (i.e., name, journal abbreviation, ISSN, eISSN). Figure 1 shows how the publications were selected. Scopus and WoS search returned 4059 and 5066 records, respectively. These records were refined by selecting: (i) “Article” and “Review” in the field “Document type, (ii) “Psychology” and “Neuroscience” in the field “Subject Area,” and (iii) “English” in the field “Language” of each database. Using this refinement, 306 and 220 papers in Scopus and WoS, respectively, met the criteria and thus two different datasets were extracted in Excel format. Then, they were merged and duplicates were removed, resulting in a dataset of 353 publications. This dataset was further manually reviewed in order to remove unrelated papers. The unrelated papers were all the manuscripts that were detected by Scopus and WoS but did not report studies on the effects of lying on memory for the original event, but rather studies either on deception detection or memory. The final dataset was composed of 70 publications (available on OSF^3^).

### Data Analysis

A plethora of software is available to analyze data with a scientometric approach (e.g., Bibliometrix R Package, BibExcel, CiteSpace, Sci, VOSviewer). For the current work, VOSviewer was used (Van Eck et al., 2010; Van Eck and Waltman, 2014); it is a free software program allowing the creation of bibliometric maps based on bibliographic data (e.g., co-authorship, keyword co-occurrence, citation, co-citation) of the reviewed publications. A performance analysis was conducted on authors, authors’ information (i.e., country, affiliated institutions), and publications information (i.e., journal, year of publication, citations) as well as a co-citation analysis on authors and documents. In addition, to identify networks between authors’ keywords in the set of publications, a cluster analysis on Keywords Co-Occurrence Network (KCN) was performed. Therefore, based on the publications resulting from the KCN, a targeted overview on the aims, methods, and critical issues related to such a pool of publications was performed.

## Results

### Performance of Countries

The number of publications on the effects of lying on memory by countries is shown in Table 1. Specifically, Table 1 shows the publications split based on the number of single (SCP) and multiple country publications (MCP) for all the countries of the dataset. The SCP refers to publications in which all authors belong to the same country, while the MCP represents publications involving authors of different countries. In the top five of the most productive countries (i.e., United States, Netherlands, United Kingdom, Belgium, Italy), the majority of publications of the leading country – the United States – included studies involving scholars of the same country, while the other countries – Netherlands, United Kingdom, Belgium, and Italy – published work that stimulated international collaboration.

**TABLE 1:** Number of publications on the effects of lying on memory by country.

Country	Number of Publications	SCP	MCP
United States	28	25	3
Netherlands	19	4	15
United Kingdom	10	4	6
Belgium	10	0	10
Italy	9	1	8
Sweden	5	3	2
France	3	3	0
China	1	1	0
Canada	1	0	1
Germany	1	1	0
Israel	1	1	0
Russia	1	1	0

*SCP indicates Single Country Publications and MCP, Multiple Country Publications. Publications having the first author affiliated to different countries were counted as many times as countries of affiliations.*

**FIGURE 2:**
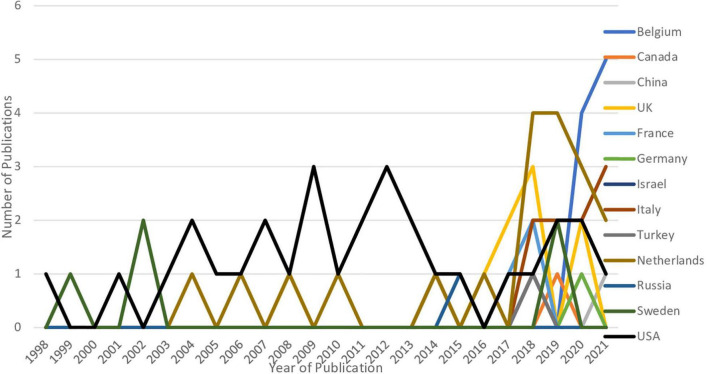
Number of publications by country and by year for all the twelve countries and total number of publications by year from 1998 to 2021.

An inspection of the number of publications published by country and by year was also conducted. Figure 2 presents a graph of the publications published from 1998 to 2021 in the 12 countries of interest. As can be seen, in the last 3 years (i.e., from 2018 to 2021) literature on the effects of lying on memory has increased. As displayed in Figure 2, the main countries that published the lion’s share of articles during this recent peak were the Netherlands, Italy, and Belgium, whereas the United States – the leading country in terms of total publications – did not have a peak of publications but instead published at least one paper every year within the period from 1998 to 2021.

### Performance of Journals

Table 2 presents the number of publications on the effect of lying on memory by all the journals in the dataset with the total global citation score (TGCS). The TGCS is the number of times the publication has been cited in the database used to download the sources. We obtained the index by summing the citations of all the publications of each journal. In total, work on lying and memory has been published in 25 journals in the research areas of Psychology (*n* = 22), Criminology (*n* = 2), Criminology and Law (*n* = 1). In the top 10 most productive journals – of which 7 in the research area of Psychology, 2 in Criminology, and 1 in Criminology and Law – 72% (52/70) of the total publications reaching 68.2% of total citations (690/1012) was published. Specifically, only the leading journal (i.e., *Applied Cognitive Psychology*) published more than 10 papers on the effects of lying on memory, while three journals (i.e., *Memory, Memory and Cognition*, and *Acta Psychologica*) published from 5 to 8 papers, and the rest (i.e., *Psychology, Crime, and Law, Journal of Applied Research in Memory and Cognition, Legal and Criminological Psychology, Frontiers in Psychology, Law and Human Behavior*, and *Psychological Research*) from 2 to 4 papers. Regarding the number of citations of the top 10 most productive journals, it is noteworthy that the higher numbers of publications did not necessarily result in having a high number of citations. Indeed, 2 journals (i.e., *Applied Cognitive Psychology* and *Memory and Cognition*) reached a very good performance (i.e., more than 100 citations), 6 journals obtained a good performance (i.e., more than 50 citations), and 2 (i.e., *Frontiers in Psychology* and *Psychology, Crime, and Law*) a moderately good performance (i.e., less than 40 citations). By contrast, four journals that published only one paper reached a very good or good performance with more than 100 or 50 citations.

**TABLE 2:** Number of publications on the effects of lying on memory by source and total global citation scores (TGCS).

Journal	Number of Publications	TGCS
Acta Psychologica	5	52
American Journal of Forensic Psychology	1	1
American Journal of Psychology	2	17
Applied Cognitive Psychology	12	185
Behavioral Sciences and The Law	2	22
Brain and Cognition	1	2
Cognition	1	2
Developmental Psychology	1	120
Europes Journal of Psychology	1	4
Frontiers in Psychology	3	13
Journal of Applied Research in Memory and Cognition	4	58
Journal of Experimental Psychology-General	1	18
Journal of Experimental Psychology-Learning, Memory, and Cognition	1	2
Journal of General Psychology	1	0
Law and Human Behavior	3	51
Legal and Criminological Psychology	4	69
Memory	8	57
Memory and Cognition	7	116
Psychological Research	2	53
Psychological Science	1	73
Psychology and Law	1	0
Psychology, Crime, and Law	4	36
Psychonomic Bulletin and Review	1	48
Quartery Journal of Experimental Psychology	2	10

### Performance of Institutions

An analysis on the number of publications on the effects of lying on memory by institutions of the first author was performed. This analysis was performed by considering the country, the total global citation scores (TGCS), and the total citations per year (TCpY). Table 3 shows the results for all the institutions of the dataset. We detected 35 institutions which were mainly based in the United States (*n* = 20). However, the majority of the top 10 most productive institutions are located in Europe (*n* = 7), with a minority in the United States (*n* = 3). In particular, the leading institutions – having the highest number of publications and TGCS and one of the highest TCpY (19, 199, 11.71, respectively) – was Maastricht University (the Netherlands), followed by the Catholic University of Leuven (Belgium) and the University of Bari Aldo Moro (Italy) with a high number of publications (10 and 8, respectively) and high or good TGCS (31 and 32, respectively) and TCpY (15.5 and 8.00, respectively). The rest of the 10 most productive institutions – from the United Kingdom (i.e., City University of London), United States (Kent State University, Claremont Graduate University, and Central Washington University), France (University of Lille), and Sweden (Stockholm University) – had an average number of publications (from 5 to 3), but high or good TGCS (26–61) and TCpY (15.75–2.73). Interestingly, some institutions that published only one article obtained high TGCS and TCpY, i.e., Gustavus Adolphus College (United States, publications: 1, TGCS: 120, and TCpY: 5.22), and Montana State University (United States, publications: 1, TGCS: 45, TCpY: 15.00).

**TABLE 3:** Number of publications on the effects lying on memory by institution, country, number of publications, total global citation scores (TGCS), and total citations per year (TCpY).

Institutes	Country	Publications	TGCS	TCpY
Ball State University	UNITED STATES	1	25	1.79
Bar-Ilan University	ISRAEL	1	12	3.00
Bilkent University	TURKEY	1	3	0.43
Brandeis University	UNITED STATES	2	3	0.43
Catholic University of Leuven	BELGIUM	10	31	15.5
Maastricht University	NETHERLANDS	19	199	11.71
Central Washington University	UNITED STATES	3	34	3.78
City University of New York	UNITED STATES	1	3	0.75
Claremont Graduate University	UNITED STATES	3	60	6.67
Emporia State University	UNITED STATES	1	17	8.50
Friedrich Schiller University Jena	GERMANY	1	1	0.50
Gustavus Adolphus College	UNITED STATES	1	120	5.22
Kennesaw State University	UNITED STATES	1	0	0
Kent State University	UNITED STATES	4	161	8.05
Lomonosov Moscow State University	RUSSIA	1	0	0
Louisiana State University	UNITED STATES	1	19	3.17
City University of London	UNITED KINGDOM	5	68	13.6
Gothenburg University	SWEDEN	2	15	5.00
McGill University	UNITED STATES	1	4	1.34
Montana State University	UNITED STATES	1	45	15.00
Pepperdine University	UNITED STATES	1	30	1.76
Saint Martin’s University	UNITED STATES	1	5	0.84
Southern Connecticut State University	UNITED STATES	1	0	0
Southern New Hampshire University	UNITED STATES	2	17	0.95
Stockholm University	SWEDEN	3	60	2.73
Tianjin Normal University	CHINA	1	0	0
University of Aberdeen	UNITED KINGDOM	2	59	4.54
University of Bari Aldo Moro	ITALY	8	32	8.00
University of California Davis	UNITED STATES	1	18	2.57
University of Denver	UNITED STATES	1	0	0
University of Lille	FRANCE	3	63	15.75
University of North Carolina	UNITED STATES	1	13	1.45
University of Portsmouth	UNITED KINGDOM	4	26	6.50
University of Rome La Sapienza	ITALY	1	2	1.00
Wesleyan University	UNITED STATES	1	15	1.25

*Publications having the first author affiliated to different institutions were counted as many times as institutions of affiliations.*

### Performance of Authors

The analysis on the performance of authors was performed. The top 10 most productive authors based on the number of publications are listed in Table 4. In the table, the number of publications for each author is presented by considering the number of single, multi, and first-authored publications. Overall, these authors were involved in 48.57% (*n* = 34) of the total publications on the effects of lying on memory of which only 5 were single-authored. The first two authors – Otgaar, H., and Mangiulli, I. – of the top 10 most productive authors had more than 10 publications (15 and 13, respectively), followed by Van Oorsouw, K., Zaragoza, M.S. and Battista, F. with more than 5 publications (8, 7, and 6, respectively) and the rest of the list with 4 or 3 publications. Of interest, more than half of the most productive authors’ own publications, except Polage, D. C., were multi-authored publications.

**TABLE 4:** Number of single, multi, and first-authored publications on the effects of lying on memory by the 10 most productive authors.

Author	Total Publications	Single-Authored	Multi-Authored	First-Authored	Percentage (%)
Ackil, J.K.	3	0	3	2	4.29
Battista, F.	6	0	6	4	8.57
Bylin, S.	3	1	2	2	4.29
Harvey, A.C.	3	0	3	3	4.29
Mangiulli, I.	13	0	13	5	18.57
Otgaar, H.	15	0	15	5	21.43
Pezdek, K.	3	0	3	2	4.29
Polage, D.C.	4	4	0	1	5.71
Riesthuis, P.	4	0	4	3	5.71
Romeo, T.	3	0	3	2	4.29
Van Oorsouw, K.	8	0	8	4	11.43
Zaragoza, M.S.	7	0	7	1	10.00

*Percentage was calculated by considering all authors’ contributions in the revised publications. Moreover, the list consists of 12 authors because Ackil, J.K., Pezdek, K., and Romeo, T. had the same performance.*

**FIGURE 3:**
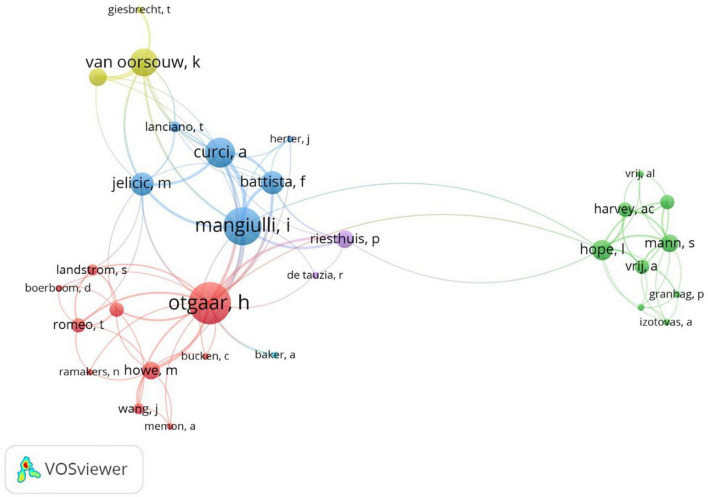
The co-authorship network.

To verify the co-occurrence relationships among authors, a co-authorship analysis was run. Figure 3 shows the co-authorship network with each node representing an author and the lines corresponding to the collaborative actions. The larger the node, the higher the number of the author’s publications. Also, the thicker the line, the more collaboration exists between authors. The network demonstrated different research collaborations among scholars, i.e., 98 collaborative actions. In particular, as shown in Figure 3 main communities were detected and the authors that predominate the network in terms of both number of publications and collaborative actions were Otgaar, H., Mangiulli, I., Van Oorsouw, K., Curci, A., and Battista, F.

### Document Citation Analysis

Table 5 presents the 10 most cited documents. Specifically, the title of the publication and the number of citations are shown for each publication. The first most cited publication was Ackil and Zaragoza (1998) with more than 100 citations, followed by Zaragoza et al. (2001) with almost 100 citations. Only one publications – Chrobak and Zaragoza (2008) – received more than 50 citations, while the rest of the top 10 most cited articles, i.e., Christianson and Bylin (1999), Pickel (2004), Polage (2004), Van Oorsouw and Merckelbach (2004), Van Oorsouw and Merckelbach (2006), Pezdek et al. (2007), Otgaar and Baker (2018) obtained from 38 to 27 citations.

**TABLE 5:** The top 10 cited articles.

Authors	Title of the Publication	Citations
Ackil and Zaragoza, 1998	Memorial consequences of forced confabulation: Age differences in susceptibility to false memories	130
Zaragoza et al., 2001	Interviewing Witnesses: Forced Confabulation and Confirmatory Feedback Increase False Memories	98
Chrobak and Zaragoza, 2008	Inventing stories: Forcing witnesses to fabricate entire fictitious events leads to freely reported false memories	56
Christianson and Bylin, 1999	Does simulating amnesia mediate genuine forgetting for a crime event?	38
Pickel, 2004	When a lie becomes the truth: The effects of self-generated misinformation on eyewitness memory	32
Van Oorsouw and Merckelbach, 2004	Feigning amnesia undermines memory for a mock crime	32
Pezdek et al., 2007	Interviewing witnesses: The effect of forced confabulation on event memory	31
Polage, 2004	Fabrication deflation? The mixed effects of lying on memory	30
Van Oorsouw and Merckelbach, 2006	Simulating amnesia and memories of a mock crime	28
Otgaar and Baker, 2018	When lying changes memory for the truth	27

### Author Citation Analysis

An analysis on the number of citations by authors was performed. Table 6 reports the 10 most cited authors. In the table, the number of citations and the Total Link Strength of the co-authorship links with other authors (TLS) for each author are presented. The first most cited author was Zaragoza, M.S. with more than 300 citations, followed by Ackil, J.K., and van Oorsouw, K. with more than 100 citations and Otgaar, H., with almost 100 citations. The rest of the top 10 most cited authors, i.e., Chrobak, Q. M., Polage, D. C., Bylin, S., Pzedek, K., Christianson, K. A., Mangiulli, I. reported more than 50 citations (from 65 to 55 citations), except Mangiulli, I. that had 36 citations. Interestingly, the majority of these authors reported a low TLS score, except for Otgaar, H., and Mangiulli, I. (15 and 13, respectively).

**TABLE 6:** The 10 most cited authors.

Author	Number of Citations	TLS
Ackil, J. K.	192	2.00
Bylin, S.	61	2.00
Christianson, K. A.	55	2.00
Chrobak, Q. M.	65	2.00
Mangiulli, I.	36	13.00
Otgaar, H.	98	15.00
Polage, D. C.	64	0.00
Pzedek, K.	60	3.00
Van Oorsouw, K.	114	8.00
Zaragoza, M. S.	303	6.00

*The table shows the number of citations and the Total Link Strength (TLS) by author.*

### Document Co-citation Analysis

A document co-citation analysis (DCA) gives a network of co-cited publications by providing the knowledge base of these publications (Li et al., 2017). Specifically, this analysis represents how many times two publications have been jointly cited by other publications (Small, 1973; Zhong et al., 2009). Thus, because the references cited in manuscripts provide the knowledge base of such publications, DCA objectively identified the underpinning knowledge base of the selected publications (Li et al., 2017). The analysis demonstrated a document co-citation network containing 164 nodes and 2921 links. Each node represents a publication that is identified by the first author name and the publication year, every link is a co-citation relationship between the two corresponding publications, and the size of the node is the co-citation frequency of the publications. As shown in Table 7, the top 10 publications most co-cited were: Ackil and Zaragoza (8 co-citations), Vieira and Lane (2013) (6 co-citations), Zaragoza et al. (2001) (6 co-citations), Van Oorsouw and Merckelbach (2004), Chrobak and Zaragoza (2008), Sun et al. (2009) (all 5 co-citations); Johnson et al. (1993), Anderson and Green (2001), Polage (2012), Walczyk et al. (2014) (all 4 co-citations).

**TABLE 7:** The top 10 co-cited articles.

Authors	Title of the Publication	Co-citations
Ackil and Zaragoza, 1998	Memorial consequences of forced confabulation: Age differences in susceptibility to false memories	8
Vieira and Lane, 2013	How you lie affects what you remember	6
Zaragoza et al., 2001	Interviewing Witnesses: Forced Confabulation and Confirmatory Feedback Increase False Memories	6
Chrobak and Zaragoza, 2008	Inventing stories: Forcing witnesses to fabricate entire fictitious events leads to freely reported false memories	5
Van Oorsouw and Merckelbach, 2004	Feigning amnesia undermines memory for a mock crime	5
Sun et al., 2009	Does feigning amnesia impair subsequent recall?	5
Polage, 2012	Fabrication inflation increases as source monitoring ability decreases	4
Anderson and Green, 2001	Suppressing unwanted memories by executive control	4
Walczyk et al., 2014	A social-cognitive framework for understanding serious lies: Activation-decision-construction-action theory	4
Johnson et al., 1993	Source monitoring.	4

### Author Co-citation Analysis

**FIGURE 4:**
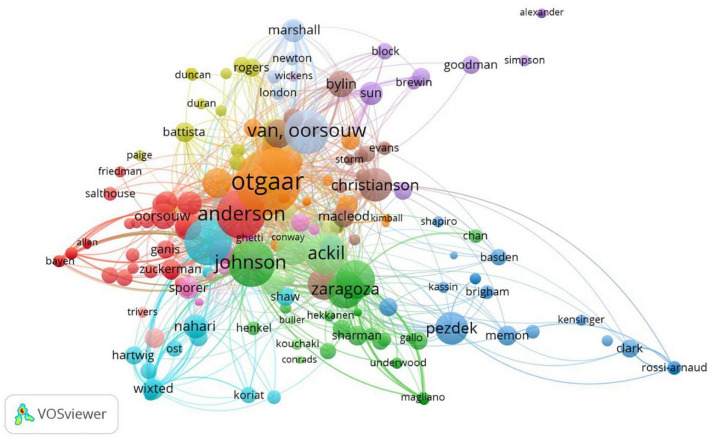
Authors’ co-citation network.

An author co-citation analysis (ACA) counts the frequency with which any publication of an author is co-cited with another author in the references of citing documents and, therefore, provides the relationships among authors whose publications are cited in the articles (Bayer et al., 1990). Figure 4 displays the author co-citation network, containing 462 nodes and 15518 links. The size of nodes corresponds to the number of authors’ co-citations, while the links refer to the indirect cooperative relationships on the basis of the co-citation frequency. The most 10 cited authors were Otgaar, H. (64 co-citations, 306 links, and TLS 58.78), Anderson, M. C. (36 co-citations, 383 links, and TLS 35.07), Vrij (34 co-citations, 265 links, and TLS 32.33), Johnson, M. K. (33 co-citations, 355 links, and TLS 32.02), Van Oorsouw, K. (30 co-citations, 251 links, and TLS 29.14), Ackil, J. K. (27 co-citations, 339 links, and TLS 26.64), Polage, D. C. (24 co-citations, 254 links, and TLS 23.51), Loftus, E.F. (23 co-citations, 319 links, and TLS 22.44), Zaragoza, M. S. (21 co-citations, 298 links, and TLS 20.69), and Christianson, S. A. (17 co-citations, 307 links, and TLS 16.86).

### Cluster Analysis

**FIGURE 5:**
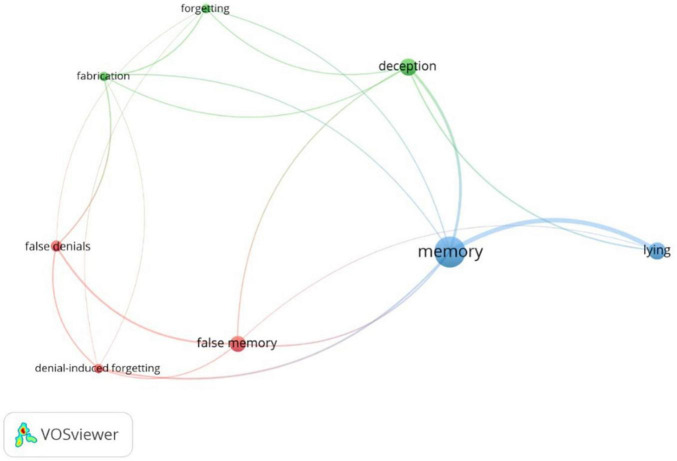
The authors’ keywords network.

**FIGURE 6:**
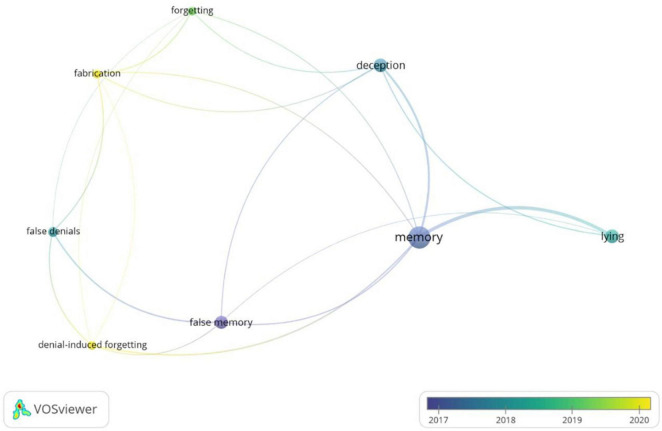
The authors’ keywords network considering the influence of time.

With the aim to investigate the semantic data of the published studies, a cluster analysis on the Keywords Co-Occurrence Network (KCN) was carried out. A cluster analysis is a statistical technique that permits to identify the relative degree of correlation of terms and classify a large pool of semantic data pertaining to a specific theme into the same group and data pertaining to a different theme into other groups. In this way, it is possible to identify the themes, trends, and association of a corpus of studies (Olawumi and Chan, 2018; Caffò et al., 2020). The Keyword Co-occurrences (KC) analyses the presence, frequency, and proximity of keywords that – due to their topic – are similar to each other in order to highlight the occurrence of a keyword in at least two papers of a dataset. Therefore, the KCN provides a network composed of different clusters related to each other with a different degree of strength. The strength, called Total Link Strength (TLS), is expressed by a numerical value corresponding to the amount of publications in which two keywords are simultaneously used. The higher this value, the higher is the strength (Radhakrishnan et al., 2017). By using VOSViewer (Van Eck and Waltman, 2014), we used the following parameters for our KNC analysis: “Authors’ Keywords,” “Fractional counting” (i.e., the links’ weight is fractionalized), and 1 as the minimum number of co-occurrences. Therefore, 128 keywords, 16 clusters, 383 links, and a TLS of 109.50 were identified (Supplementary Figure 7). However, in order to have a clear picture of the most used keywords, we refined the analysis by adding the option to run the analysis with a co-occurrences threshold (i.e., the minimum number of occurrences of a keyword to enter the network) set on the default value suggested by VOSViewer, i.e., 5. After this selection, 3 clusters, 19 links, and a TLS of 29.00 were detected (Figure 5 and Table 8). Specifically, the analysis identified the following clusters: Cluster 1 consisted of “denial-induced forgetting,” “false denials,” and “false memory”; Cluster 2 included “deception,” “fabrication,” and “forgetting”; Cluster 3 was composed of “lying,” and “memory.” Table 8 shows the three clusters with the corresponding keywords, occurrences, links, TLS. For Cluster 1, the keyword “false memory” was the most cited (9 occurrences, 5 links, TLS of 6), followed by “false denials” (both 6 occurrences, 4 links, TLS of 5), and “denial-induced forgetting” (5 occurrences, 5 links, TLS of 5). For Cluster 2, “deception” was the most cited keyword (10 occurrences, 5 links, TLS of 8), followed by “fabrication” (5 occurrences, 5 links, TLS of 5), and “forgetting” (both 5 occurrences, 5 links, TLS of 4). Finally, for Cluster 3, the keyword “memory” was the most cited (20 occurrences, 6 links, TLS of 16), followed by “lying” (10 occurrences, 3 links, TLS of 9). In addition, we run an additional analysis in order to check the influence of time on cluster analysis (Figure 6). The time range of clusters (i.e., years in which the keywords in the cluster have been frequently used) was the following: Cluster 1, 2014–2020; Cluster 2, 2017–2020, and Cluster 3, 2017–2018.

**TABLE 8:** Keywords and their occurrences, links, and total link strength (TLS) for the three detected clusters.

Cluster	Keywords	Occurrences	Links	TLS
1	Denial-Induced Forgetting	5	5	5
1	False Denial	6	4	5
1	False memory	9	5	6
2	Deception	10	5	8
2	Fabrication	5	5	5
2	Forgetting	5	5	4
3	Lying	10	3	9
3	Memory	20	6	16

### Selected Overview

Based on the cluster analysis, a selected overview was conducted on the publications using the eight keywords detected in the KCN which amounted to a total of 33 publications. The review was done in order to identify the following studies’ information: (i) Main aim, (ii) kind of deceptive strategy employed, (iii) type of stimulus, (iv) type of memory task, (v) significance of the results, and (vi) limitations. We decided to review this information in order to provide information on the main questions investigated in the considered publications by highlighting the adopted methods and by pointing out their limitations. The information “Main aim,” “Deceptive strategy,” “Type of stimulus,” “Type of memory task,” and “Limitations” was simply extracted from each publication and “nd” (i.e., no data) was assigned if no information was detected, while for the information “Significance of the results,” we assigned “Yes” if the authors found a statistically significant effect of deception on memory and “No” if the authors did not find any statistically significant effect.

Table 9 shows the authors with the year of publication and the title of the study together with the above-mentioned information (i.e., aim, deceptive strategy, stimulus, memory task, significance of the results, and limitations). With regards to the main goals of the 33 selected publications, for 21 publications the investigated issue was understanding which effects each deceptive strategy (i.e., false denials, feigned amnesia, and fabrication) might have on memory. In addition, 6 publications simultaneously investigated the effects of different deceptive strategies on memory with 5 examining the strategies of both false denials and fabrication on memory, while 1 examined the strategies of both false denials and feigned amnesia on memory. Furthermore, 4 publications examined how lying affected liars’ beliefs on the occurrence of false events and 1 how the act of repeatedly lying can affect liars’ memory for the original event. Finally, 1 of the selected publications aimed to survey people to examine their knowledge and beliefs on how lying affects memory, while 1 was a review on the literature on lying and memory. Regarding the type of deception studied, 21 publications took into consideration the fabrication strategy, 16 the false denials strategy, and 6 the feigned amnesia strategy. Concerning the type of stimulus depicting the event which participants had to lie on, 14 publications used a video, 8 asked participants to perform actions, 4 events list, and 3 showed pictures. Each of all the other stimuli (i.e., narrative, Virtual Reality Scenes, biography, word lists) was used in only 1 publication. With respect to the type of memory task used to verify whether lying had an effect on memory, the principal memory tasks used were free recall and source monitoring (6 publications for both). However, scholars adopted also cued recall, beliefs rating for the memory of false events (i.e., both in 4 publications) or a recognition task (i.e., 3 publications). Interestingly, many publications adopted combined measures of memory. Specifically, 4 studies used both recognition task and cued recall, 3 publications both free and cued recall, and 1 publication both free recall and recognition task. Combining the type of stimulus and memory task, studies using a video as a stimulus mainly used cued recall (i.e., 9 publications) and free recall (i.e., 6 publications) as a memory task. However, the majority of these studies used a combined measure for both types of tasks (i.e., cued recall: 9 publications of which 7 combined with another memory task; free recall: publications of which 4 combined with another memory task). With regards to studies using actions, the free recall, source monitoring, and recognition tasks were used with the same frequency (i.e., 2 publications for each). By contrast, in studies adopting event lists, the most used memory task was the beliefs rating (i.e., 3 publications), while in the case of studies using pictures, the most used task was source monitoring (i.e., 2 publications). Moreover, with regards to the significance of the results achieved in the selected publications, it is noteworthy to underline that all publications, except 2, reported a statistically significant effect of lying on memory. In particular, the majority of the studies found an undermining memory effect of lying by using different stimuli, memory tasks, and by considering the three deceptive strategies. Finally, concerning the main limitations addressed by authors in their publications, the main issues were: (i) the generalizability of the achieved findings for real situations (16 publications), (ii) lack of a direct control of the manipulation adopted (e.g., different type of lying, cognitive load necessary to lie) (10 publications), (iii) need of additional measures (e.g., memory tasks, stimuli, physiological task) (4 publications), (iv) not studied mechanisms underpinning the effects of lying on memory (3 publications), (v) need of investigating different kinds of deception simultaneously (2 publications), (vi) need of considering different perspective taking (1 publication), and (vii) no replication of prior findings (1 publication). In addition, the only review on the topic (i.e., Otgaar and Baker, 2018) underlined a lack of studies on this line of research. In addition, combining the type of stimulus adopted to the limitations reported by the authors, we found that in studies adopting more naturalistic stimuli^4^ (i.e., stimuli that employ rich, multimodal dynamics) the main limitations were: (i) the generalizability of the achieved findings for real situations (12 publications), (ii) lack of a direct control of the manipulation (9 publications), (iii) need of additional measures (e.g., memory tasks, stimuli, physiological task) (4 publications), (iv) not studied mechanisms underpinning the effects of lying on memory (1 publication), (v) need of investigating different kinds of deception simultaneously (2 publications), (vi) need of considering different perspective taking (1 publication). However, for studies with more artificial stimuli (stimuli that do not employ the rich, multimodal dynamics) the main limitations were: (i) the generalizability of the achieved findings for real situations (4 publications), (ii) lack of a direct control of the manipulation (1 publication), and (iii) not studied mechanisms underpinning the effects of lying on memory (2 publications).

**TABLE 9:** Information of the selected publications for the critical review.

Authors and Year	Title	Aim	Deceptive Strategy	Stimulus	Memory Task	Significance of the Results	Limitations
Battista et al., 2021a	What Can We Remember After Complex Denials? The Impact Of Different False Denials On Memory	Test the effects of two different cognitively demanding false denials on memory	False Denials	Video	Recognition and Cued Recall	Yes	- Generalization of the findings for real situations - Lack of test for the cognitive manipulation - Only one strategy - Only witness perspective
Battista et al., 2020	The Effects Of Repeated Denials And Fabrication On Memory	Test the effect of repeated lying on memory	False Denials and Fabrication	Video	Recognition and Cued Recall	Yes	- Generalization of the findings for real situations - Feigned Amnesia not tested - Mechanisms Underpinning not studied
Battista et al., 2021c	Do Liars Really Remember What They Lied Upon? The Impact Of Fabrication On Memory	Test the effects of two different cognitively demanding fabrication on memory	Fabrication	Video	Recognition and Cued Recall	Yes	- Memory task used in the pilot study different from the one of the main study - No replication of prior findings on beliefs’ rating
Battista et al., 2021b	The Role Of Executive Functions In The Effects Of Lying On Memory	The role of individual executive functions resources in the effects of lying on memory	False Denials and Fabrication	Video	Recognition and Cued Recall	Yes	nd
Besken, 2018	Generating Lies Produces Lower Memory Predictions and Higher Memory Performance Than Telling the Truth: Evidence for a Metacognitive Illusion	Test a lie-generation manipulation on both actual and predicted memory performance	Fabrication	General Events List	Free Recall	Yes	- Generalization of the findings for real situations
Chrobak and Zaragoza, 2012	When Forced Fabrications Become Truth: Causal Explanations and False Memory Development	Test how the formation of false memory can happen due to fabrication	Fabrication	Video	Free Recall	Yes	- Generalization of the findings for other deceptive strategies
Gombos et al., 2012	Forced confabulation affects memory sensitivity as well as response bias	Test how forced confabulation affect memory for the actual event	Fabrication	Video	Cued Recall	Yes	nd
Harvey et al., 2017a	A Stability Bias Effect Among Deceivers	Test the memory for the event and stability bias	Fabrication	Action	Free Recall	Yes	- Generalization of the findings for real situations
Harvey et al., 2017b	Deception and Decay: Verbal Lie Detection as a Function of Delay and Encoding Quality	Test how encoding quality and retention interval affect memory after lying	Fabrication	Video and Action	Cued Recall	Yes	- Generalization of the findings for real situations - Cognitively simple situation of lying
Li and Liu, 2021	Involvement Modulates the Effects of Deception on Memory in Daily Life	Test DIF effect with a daily life paradigm	False Denial	Action	Source Monitoring	Yes	- Generalization of the findings due to the sample composition
Mangiulli et al., 2019a	Do Reminders Of The Crime Reverse The Memory-Undermining Effect Of Simulating Amnesia?	Test whether reminders about the lied event reverse the memory-undermining effect of feigned amnesia	Feigned Amnesia	Video	Free and Cued Recall	Yes	- Generalization of the findings for real situations - Lack of test for the manipulation adopted - Not adopted different memory tasks
Mangiulli et al., 2019b	Retrieval-Induced Forgetting in the Feigning Amnesia for a Crime Paradigm	Test whether retrieval-induced forgetting underlies the memory-undermining effect of feigned amnesia	Feigned Amnesia	Video	Free Recall	Yes	- Generalization of the findings for real situations - Lack of test of the manipulation adopted
Mangiulli et al., 2018	Feigning Amnesia Moderately Impairs Memory for a Mock Crime Video	Test the effects of feigned amnesia adopting a video	Feigned Amnesia	Video	Free and Cued Recall	Yes	- Generalization of the findings for real situations - No comparison of different stimuli - Not clear whether participants mixed deceptive strategies - Need to use questionnaire to assess cognitive functions
McWilliams et al., 2014	Memory For Child Sexual Abuse Information: Simulated Memory Error And Individual Differences	Test the effects of simulated memory (i.e., false denied or fabricated details) error on memory for CSA information	False Denials and Fabrication	Narrative	Free and Cued Recall	Yes	- Generalization of the findings for real situations - Lack of control for the adopted memory tasks
Otgaar and Baker, 2018 *	When Lying Changes Memory For The Truth	Review literature on the effects of lying and memory	False Denials, Feigned Amnesia, and Fabrication	nd	nd	nd	- Lack of studies
Otgaar et al., 2020	The Impact Of False Denials On Forgetting And False Memory	Test the effect of false denials on forgetting and false memory formation	False Denials	Word Lists	Free Recall or Source Monitoring	Yes	- Generalization of the findings for real situations
Otgaar et al., 2016	Denial-Induced Forgetting: False Denials Undermine Memory, But External Denials Undermine Belief	Test the effects of false denials on memory	False Denials	Pictures and Video	Source Monitoring	Yes	nd
Otgaar et al., 2018	Forgetting Having Denied: The Amnesic Consequences Of Denial	Test the DIF effect for different memory tasks	False Denials	Video	Free Recall or Source Monitoring	Yes	- Generalization of the findings for real situations
Paige et al., 2019	Influence Of Age On The Effects Of Lying On Memory	Test the effects of lying considering the role of cognitive control	False Denials and Fabrication	Action	Recognition	Yes	- Lack of test of the measure adopted
Paige et al., 2020	Evaluating Heart Rate Variability As A Predictor Of The Influence Of Lying On Memory	Test whether heart rate variability is involved in the effects of lying one memory	False Denials and Fabrication	Action	Recognition	Yes	- Not adopted different individual differences measures - Need to use physiological measures
Pezdek et al., 2007	Interviewing Witnesses: The Effect Of Forced Confabulation On Event Memory	Test whether forced confabulation can increase the recall of details never occurred	Fabrication	Video	Cued Recall	Yes	- Generalization of the findings for real situations
Polage, 2017	The Effect of Telling Lies on Belief in the Truth	Test the effects of lying on beliefs in the memory for the truth	Fabrication	Events List	Belief Rating	Yes	- Need of more control on the manipulation adopted - Mechanisms Underpinning not studied
Polage, 2012	Fabrication Inflation Increases As Source Monitoring Ability Decreases	Test the effects of lying on beliefs in a false childhood event	Fabrication	Events List	Belief Rating	Yes	- Mechanisms Underpinning not studied
Polage, 2018	Liar, Liar: Consistent Lying Decreases Belief In The Truth	Test the effects of lying on beliefs in participants’ childhood events	False Denials	Events List	Belief Rating	Yes	- Lack of direct measure of memory for the event - Lack of control of the manipulation adopted
Riesthuis et al., 2021a	Public Beliefs On The Relationship Between Lying And Memory	Survey beliefs of students and general public on the effects of lying and memory	False Denials, Feigned Amnesia, and Fabrication	nd	nd	nd	nd
Riesthuis et al., 2021b *	Registered Report: The Effects Of Incentivized Lies On Memory	Test the effect of deceptive behavior on memory	Fabrication	Action	Cued Recall	nd	nd
Riesthuis et al., 2020	Adopting A Fictitious Autobiography: Fabrication Inflation Or Deflation?	Test whether adopting a fictitious biography make participants believe in the fake autobiography	Fabrication	Biography	Belief Rating	no	- Generalization of the findings
Romeo et al., 2019	The Impact Of Lying About A Traumatic Virtual Reality Experience On Memory	Test the effects of lying in a virtual reality paradigm	False Denials and Fabrication	Virtual Reality Scenes	Source Monitoring	Yes	nd
Romeo et al., 2018	The Memory-Impairing Effects Of Simulated Amnesia For A Mock Crime	Test the memory undermining effect for crime-related details	False Denials and Feigned Amnesia	Action	Source Monitoring	Yes	- Generalization of the findings for real situations - Lack of control on the manipulation adopted
Rossi-Arnaud et al., 2020	Long-Lasting Positive Effects Of Collaborative Remembering On False Assents To Misleading Questions	Test the effects of collaborative remembering on the recall of self-generated details	Fabrication	Video	Free Recall and Recognition	No	- Need of different manipulations
Van Oorsouw and Giesbrecht, 2008	Minimizing Culpability Increases Commission Errors In A Mock Crime Paradigm	Test whether minimizing culpability undermines memory for the original event lied upon	Fabrication	Action	Free Recall	Yes	- Need of extra check on the manipulation adopted
Vieira and Lane, 2013	How You Lie Affects What You Remember	Test how false denials affects memory	False Denials	Pictures	Source Monitoring	Yes	- Generalization of the findings for real situations
Vo et al., 2021	How Deception And Believability Feedback Affect Recall	Test whether fabricating details and receiving believability feedback impacts memory	Fabrication	Pictures	Recognition	Yes	- Generalization of the findings for real situations - Need of extra check on the manipulation adopted

*Due to the nature of both the studies (i.e., review and survey), it was not possible to report some of the information displayed in the table. “nd” means “no data.”

## Discussion

The aim of the present study was to perform a scientometric analysis and provide a selected overview of literature about the effects of lying on memory. Our principal reason for undertaking such analysis was to provide a comprehensive picture of the state of the art on this topic and identify needs for future research topics. We performed a literature search by using Scopus and Web of Science and we collected 70 publications from 1998 to 2021 (October 21st). Our results showed the following.

To begin with, a performance analysis was conducted on countries, journals, institutions, and authors’ performance. The United States was the leading country in terms of the amount of publications on the effects of lying on memory followed by the Netherlands. Most publications from the United States were single country publications suggesting that they were not the result of international collaborations. By contrast, publications from the Netherlands – and the other countries (i.e., Belgium, United Kingdom, Italy) – were mainly multilab publications, thus demonstrating that this line of research encouraged collaborations among scholars of different countries. It is interesting to mention that when we further investigated countries’ performance, we found that this index is strictly related to the year of publications. That is, we found that the United States published a high number of publications from 1998 to 2014 (24/28)^5^, while a peak of publications in recent years, specifically from 2018 to 2021, was registered for the Netherlands, Italy, and Belgium (13/19, 9/9, 9/10, respectively)^6^. Therefore, this seems to suggest that these last three countries were the ones that recently were mostly involved in the investigation of the effects of lying on memory by strengthening international collaborations. In addition, these results underline that a gap between the United States and European countries (such as the Netherlands, Italy, and Belgium) is increasing in the last years resulting in a leading role of the European countries in this line of research.

In order to understand a possible reason for these results, we checked if this shift in countries’ performance could correspond to a switch of attention in the deceptive strategy investigated and whether authors mentioned in their manuscript a reasoning why they were interested in a specific deceptive strategy. Most studies (70%) conducted in the United States aimed to investigate the effects of fabrication on memory, while European countries also conducted research on the effects of false denials and feigning amnesia (51% in total, 23% false denials, 28% feigned amnesia). Consequently, the peak of publications from 2018 to 2021 suggests that the shift in countries was also a shift in research into the memory effects of different strategies of deception. Interestingly, the general reasoning reported by the authors to examine the effects of deceptive strategies on memory was similar in the majority of the publications regardless of the country. Indeed, in around 70% of publications, the authors argued to be interested in reproducing the real legal situations in which people deceive in an experimental setting. This argumentation was also provided in publications from American and European countries and both before and during the peak of publications (2018–2021).

However, when we looked specifically at the legal cases provided as examples in which different kinds of deception can occur, we found different reasons based on the type of deception investigated. In particular, 24 studies on fabrication referred to legal cases in which interviewers used suggestive tactics forcing the interviewee (i.e., witness, suspect, or victim) to provide information (i.e., lie by fabricating). Specifically, in several of these studies, these cases referred to situations in which children were interviewed and pressured to provide information. In contrast to this, in all publications on feigning amnesia, the authors examined this deceptive strategy because they related it to cases in which offenders use this strategy in order to obstruct investigations and interfere with legal proceedings. Finally, the main reason in publications on false denials (17 publications) was that victims of sexual abuse sometimes falsely deny being abused because they do not want to disclose a traumatic event (e.g., sexual abuse). Specifically, while presenting examples of real cases, scholars mostly reported situations in which children who are victims of sexual abuse deny such abuse during the very first interviews and come forward with the truth in later interviews. These different reasons provide a plausible explanation for the interests of different types of deception on memory across time.

Regarding the performance of journals, more than 70% of publications was published in 10 journals. The first journal was *Applied Cognitive Psychology* with more than ten publications. This is not surprising considering that this journal has specific attention for studies on autobiographical memory, the detection of deception, eyewitness memory, and statement reliability. The other journals publishing a high number of publications (5–8 publications) were *Memory, Memory and Cognition*, and *Acta Psychologica*. Again, this was not surprising based on the aim of the journals, *Memory* and *Memory and Cognition*, i.e., publishing experimental work in all the areas of memory and cognition (e.g., learning, decision making, problem solving). In addition, based on the scope of *Acta Psychologica* to publish a broader range of psychological research areas (e.g., social psychology, clinical psychology, individual differences, etc.), the high number of publications in this journal leads to the conclusion that scholars published their work on the effects of lying on memory not only in journals with a specific focus on how memory works, but also in journals promoting more general areas. This probably results from a desire of scholars to make this work visible to a higher number of people due to the strong practical implications of this work for practitioners (e.g., judges, police officers, etc.).

Moreover, we took into consideration the journals’ citation scores and we again found that *Applied Cognitive Psychology* and *Memory and Cognition* had a high performance in terms of citations with more than 100 citations. However, we additionally discovered that the number of publications in journals did not necessarily reflect the quantity of citations. Indeed, on the one hand, we found that many of the most productive journals did not have a high or good performance in terms of citations, while on the other hand, some of the journals that published only one paper reported a high performance in terms of citations. A possible explanation for this could be that the citation score strictly depends on specific indexes of the journals (e.g., the impact factor) that permit a large spreading of the publications. Therefore, a comparison of the performance among journals based on the number of citations might not provide accurate information on journals’ productivity.

Concerning the institutions’ performance, the analysis showed that – even if more than half of the institutions are based in the United States – the most productive institutions were from Europe, with Maastricht University (the Netherlands) being the most productive together with the Catholic University of Leuven (Belgium) and the University of Bari Aldo Moro (Italy). This is in line with our results on countries’ performance by time showing that in the last years the geographic area mainly involved in the investigations of the effects of lying on memory has shifted from the United States to Europe. Once again, the citation scores (Total Global Citation Score and Total Citation per Year) did not go at the same pace as the number of publications. Based also on the countries’ performance by time, it could be argued that the citation scores of some of the European most productive institutions were lower than other American less productive institutions because the publications from such American institutions were published many years before the European ones. This could have made such publications more detectable and citable.

Finally, regarding the analysis on performance, the one conducted on authors’ performance demonstrated that the most two prolific authors with more than 10 publications were Otgaar, H. and Mangiulli, I., followed by van Oorsouw, K., Zaragoza, M.S. and Battista, F. (8–6 publications).

When a co-authorship analysis was executed, the same authors – plus Curci, A. and except Zaragoza, M.S., – were detected to be the scholars that stimulated collaborations among scholars. This was also confirmed with the type of publications, i.e., single-author or multi-author, showing that none of the above-mentioned authors published single-author studies.

The further analysis on the frequency with which publications and authors were cited (i.e., document citation analysis and author citation analysis) and co-cited (i.e., document co-citation analysis and authors’ co-citation analysis) showed interesting results. Specifically, we first found that the most cited publications were articles published from the 1998 to 2001 (e.g., Ackil and Zaragoza, 1998; Zaragoza et al., 2001; Christianson and Bylin, 1999; Pickel, 2004; Chrobak and Zaragoza, 2008). This seems to confirm that, in general, the citations performance is related to the year of publication of the papers. That is, in general, it might be that actors (i.e., journals, institutions, countries, authors) having a higher number of citations are the ones that published work on lying and memory in the early stage of this line of research (i.e., 1998–2001). In addition, we found that some of the most co-cited documents (i.e., Johnson et al., 1993; Anderson and Green, 2001; Walczyk et al., 2014) were publications not directly investigating the effects of lying on memory. However, these publications were about memory mechanisms or frameworks that are used to explain how lying can affect memory. Thus, it is reasonable to assume that they were cited by authors in order to examine possible mechanisms underpinning the effects of deceptive strategies on memory or in order to explain the act of lying. Similarly, results on the author citations score showed that the most cited author was Zaragoza, M. S. Interestingly, the author’s co-citation analysis also highlighted that several co-cited authors were scholars (i.e., Anderson, Vrij, and Loftus) who did not carry out research specifically focused on the relationship between lying and memory, but rather focused on the two topics separately. Still, the explanation provided for the results of the DCA can fit these findings.

The cluster analysis conducted on authors’ keywords allowed us to identify the most used keywords gathering in clusters. Because our aim was to summarize the main trends within the literature on the effects of lying on memory, we set a co-occurrences threshold using the default value among those available. Hence, we distinguished three clusters and eight keywords meaning that the most representative and used keywords used in publications on the effects of lying on memory are eight and can be collapsed in three major groups. In particular, examining the detected keywords for each cluster, the first cluster identified keywords more related to the publication on the false denial strategy, the second cluster on feigned amnesia and fabrication, while the third cluster included general keywords not attributable to the investigation of a specific deceptive strategy. Hence, based on the identified clusters, it is reasonable to argue that a high number of publications are only centered on the mnemonic effects of false denials, while publications on the consequences of feigned amnesia and fabrication on memory are more related to each other.

The cluster analysis was also used to select the publications on which we conducted a further investigation. The review was done in order to better understand the main issues investigated so far and how they were investigated with the final aim to provide helpful suggestions for future research. Hence, we critically reviewed the aims, methods, and limitations of the selected studies. The combination of this information allows to have a more comprehensive understanding of the studies published so far and can be summarized as follows. Concerning the main goals addressed in the selected studies, the majority of the studies examined the effects of lying on memory by considering the effects of each deceptive strategy (i.e., false denials, feigned amnesia, and fabrication) at once, while just a few compared two strategies in only one study. Additionally, a few studies were interested in understanding how lying can change people’s beliefs in the occurrence of false events. Finally, only one study addressed whether and how repeatedly lying affects liars’ memory. In addition, the event information on which participants had to lie upon was mainly presented by using a video of a mock crime video (i.e., theft) or by asking them to perform actions (i.e., stealing objects or daily life experience). Interestingly, only the minority of studies used pictures or narratives. Moreover, the memory scores used to test a possible impairment in terms of forgetting or memory distortions (i.e., omissions and commissions) due to having lied were free recall scores or source monitoring scores.

It is noteworthy to underline that some studies used in their experiment multiple types of memory scores, like for instance both recognition and cued recall scores or cued and free recalls. All the findings observed in these studies were statistically significant and, specifically, demonstrated that overall lying on an event leads to a detrimental effect on memory. Finally, when we looked at the limitations stated by the authors in their papers, almost all the scholars indicated as a principal issue the impossibility to completely generalize the findings in real situations due to several reasons (Pezdek et al., 2007; Vieira and Lane, 2013; McWilliams et al., 2014; Harvey et al., 2017a,b; Besken, 2018; Mangiulli et al., 2018, 2019a,b; Otgaar et al., 2018, 2020; Battista et al., 2020, 2021a; Riesthuis et al., 2020; Li and Liu, 2021; Vo et al., 2021). In particular, the main reasons reported to explain this issue were (i) the use of a sample of the population (i.e., students) being not necessarily representative of the general population (ii) the stimulus adopted lacking of ecological validity. A second limitation was a lack in directly controlling the manipulation used during the lying phase (Van Oorsouw and Giesbrecht, 2008; McWilliams et al., 2014; Polage, 2017, 2018; Romeo et al., 2018; Mangiulli et al., 2019a,b; Paige et al., 2019; Battista et al., 2021a; Vo et al., 2021). That is, in several publications, the authors investigated the different kinds of deception by manipulating the cognitive effort required to lie or by adapting certain paradigms to fit with the research aim, but they did not provide any evidence of the supposed direction of the manipulation but instead just assumed it based on prior studies. Another important limitation raised was the need to test the variables of the study by using a larger pool of measures (Mangiulli et al., 2018, 2019a; Polage, 2018; Paige et al., 2020). In other words, several scholars underlined that multiple measures of the same investigated concept (i.e., memory, physiological, individual differences) should be combined. Still, a further limitation was the impossibility of these studies to provide a clear understanding of the mechanism underpinning the mnemonic consequences of lying (Polage, 2012, 2017; Battista et al., 2020). Indeed, the majority of studies carried out were not able to test which mechanisms (i.e., inhibition, retrieval-induced forgetting, source monitoring errors) can explain why lying undermines the memory for the original event but rather provided speculation about their results. Finally, some scholars also pointed out the need to take into consideration whether the perspective-taking (i.e., witness, offender, or victim) matters in the effects of lying on memory (Battista et al., 2021a) and the need also to simultaneously compare all the three strategies (i.e., false denials, feigned amnesia, and fabrication) (Battista et al., 2020, 2021a).

Taken together, our scientometric analysis has several implications. First, the general picture is that lying can adversely affect memory. This conclusion seems to be supported by the different type of stimuli and memory tasks adopted. Indeed, although studies used different materials and measures in accordance with the specific goal of the study, the recurrent finding was that there is a statistically significant difference between liars and truth-tellers in the recall of the original event and that such a recall is worst in liars than in truth-tellers. However, to truly capture this effect, meta-analytic studies should be conducted on the effect of lying on memory. Second, because the main goal of the studies was to test each strategy at once, there is also a large consensus on the idea that each strategy affects memory in its own way. This is in line with the main assumption of the Memory and Deception model (MAD; Otgaar and Baker, 2018). However, it is also interesting to underline that so far no studies have tested the effects of all the three strategies on memory simultaneously. This is surprising because in order to conclude that each strategy affects memory differently it is necessary having a direct comparison among the strategies in one single study. Third, based on the limitations that the authors stated in their papers, it is evident that further investigation requires to fill the gaps of the published studies. For instance, considering the practical implications of this work, it is important that future studies adopt a more ecological valid procedure (e.g., using emotional stimuli, Virtual Reality scenes) and include more representative samples of populations (e.g., people with different range of age). Similarly, it is necessary that future studies will try to replicate findings by considering other possible factors that matter in real situations, such the role of the liar (i.e., witness, offender, or victim) or other variables not considered so far, like the delay of time between the act of lying and the recall of the original event or which type of information is susceptible to forgetting after lying. Furthermore, an important future step is trying to collect more evidence on the possible mechanisms underpinning the effects of lying on memory.

## Concluding Remarks

We have presented the first scientometric analysis on the effects of lying on memory combined with a selected review. By adopting this combined review approach, we were able to provide relevant information on research not only in terms of countries, institutions, journals, and authors’ performance but only in terms of detection of the research themes, methodology, and limitations, and challenges for future research. Our findings showed that the (experimental) investigation of the effects of lying and memory is increasing in the last years (i.e., 2018–2021). The number of collaborative actions among scholars seems to be limited to few authors, suggesting that the research on this topic has captured the attention of a small group of researchers belonging to the same community. In addition, the selected review highlighted the need to continue investigating the topic by adopting more ecological paradigms and trying to provide information on un-answered questions.

This work provides useful information for researchers interested in investigating how lying affects memory by underlying that this line of research is an emerging field that necessitates further attention.

## Data Availability Statement

The datasets presented in this study can be found in online repositories. The names of the repository/repositories and accession number(s) can be found below: Open Science Framework: https://osf.io/xe4ty/.

## Author Contributions

FB conceived the study, conducted data searching, analyzed the data, and wrote the manuscript. HO critically revised the manuscript. Both authors contributed to the article and approved the submitted version.

## Conflict of Interest

The authors declare that the research was conducted in the absence of any commercial or financial relationships that could be construed as a potential conflict of interest.

## Publisher’s Note

All claims expressed in this article are solely those of the authors and do not necessarily represent those of their affiliated organizations, or those of the publisher, the editors and the reviewers. Any product that may be evaluated in this article, or claim that may be made by its manufacturer, is not guaranteed or endorsed by the publisher.

## Footnotes

1 Commission errors occur when people report details not experienced during the original event.

2 In addition, with the aim to gain a complete picture of the literature, we also performed a searching by using ProQuest, an online platform including a large pool of sources, such as full-text journals, articles of newspapers and magazines, and dissertations. However, no records meeting our criteria were detected with ProQuest.

3
https://osf.io/xe4ty/

4 Based on Sonkusare et al.’s (2019) definition, we have considered as naturalistic materials the video, actions, narrative, and the Virtual Reality Scenes stimuli, while the rest of the stimuli were classified as artificial. Thus, the percentage of naturalistic and artificial materials in studies on the effects of lying on memory was 75% (*n* = 24) and 25% (*n* = 9), respectively.

5 The number of publications reported between parentheses refers to the number of publications of the country from 1998 to 2017 by the total number of publications of the country.

6 The number of publications reported between parentheses refers to the number of publications of the country from 2018 to 2021 by the total number of publications of the country.
