# Supplementary figures and images for: Research on the Effects of Lying on Memory: A Scientometric Analysis and a Call for New Studies
Source: Front Psychol. 2022 Feb 24;13:837265. doi: 10.3389/fpsyg.2022.837265 (PMC8907922; doi:10.3389/fpsyg.2022.837265)

## Supplementary Data

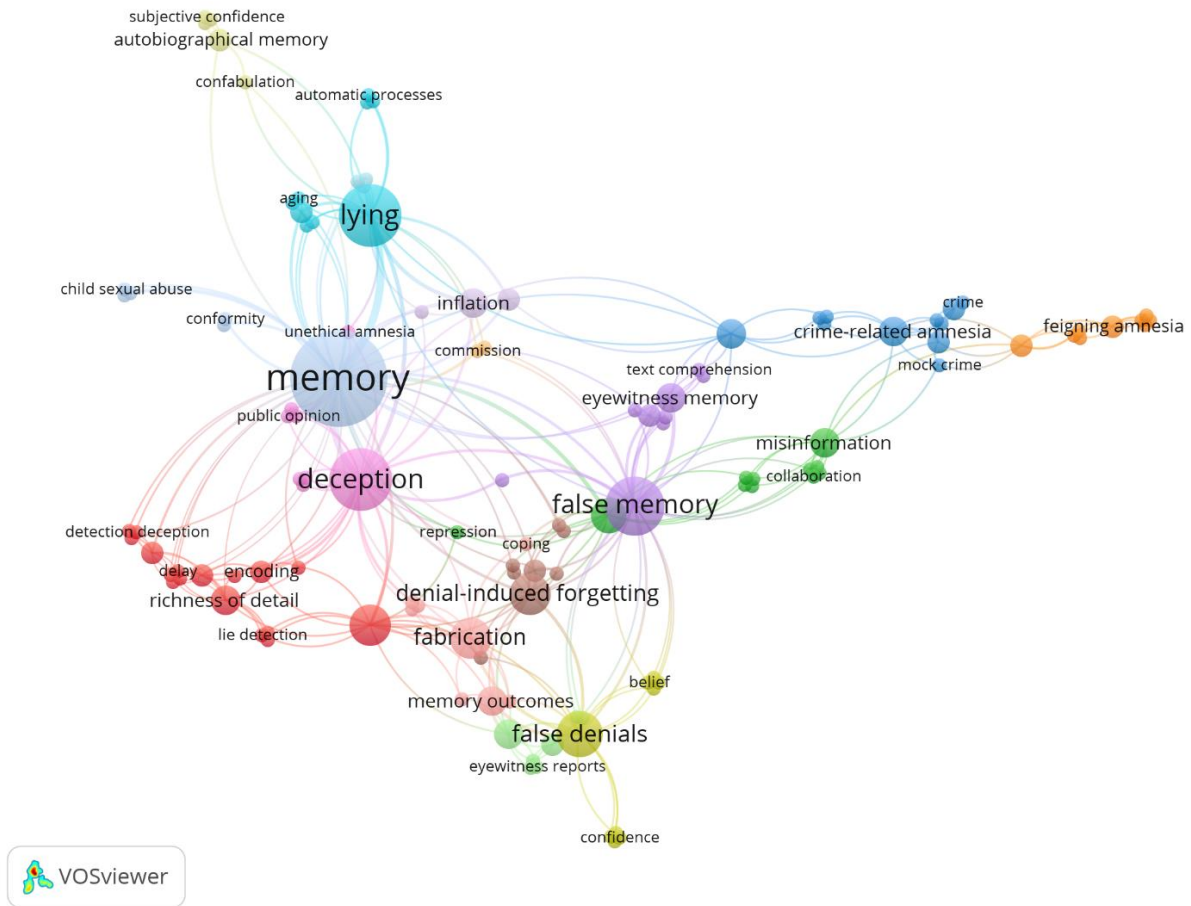

**Figure 7.** The complete Authors' Keywords Network.

Supplement: Supplementary file 1 [file Data_Sheet_1.pdf]
